# Positive and negative mood states mediated the effects of psychological resilience on emotional stability among high school students during the COVID-19 pandemic

**DOI:** 10.3389/fpsyg.2022.967669

**Published:** 2022-08-15

**Authors:** Fulei Han, Qiulin Wang

**Affiliations:** College of Physical Education, Yangzhou University, Yangzhou, Jiangsu, China

**Keywords:** psychological resilience, emotional stability, mood state, COVID-19 pandemic, high school student

## Abstract

This study investigated the parallel mediating effects of positive and negative mood states on the relationship between psychological resilience and emotional stability among first- through third-year senior high school students in China during the challenges of the COVID-19 pandemic. Of 408 questionnaires distributed from April 11 to April 22, 2022, to students at a high school located in Changzhou, Jiangsu, China, 360 were completed correctly and analyzed using a cross-sectional study design. The questionnaire included items from the modified Chinese version of the Psychological Resilience Scale, the Profile of Mood States scale, and the Eysenck Personality Questionnaire Short Scale in Chinese, the latter to assess emotional stability. The mediating effects of mood states on the relationship between psychological resilience and emotional stability were explored by using structural equation modeling and bootstrapping methods. The results indicated that psychological resilience directly affected emotional stability but also indirectly affected emotional stability through the mediating effects of positive and negative mood states. The mediating effect of negative mood states was greater than that of positive mood states. This result differs from that of research conducted prior to the pandemic, which found that compared with the damage caused by negative moods to emotional stability, positive moods more strongly promoted emotional stability. Our findings indicate that high school officials in China should consider strengthening mental health support for students who are taking courses online during home quarantine.

## Introduction

The ongoing COVID-19 pandemic has changed the way of life for many people. Home quarantine has come to be regarded as an effective way to limit the spread of the virus, making a consideration of the mental health of people in home quarantine an important focus. Indeed, mental health problems, including depression, insomnia, fear, and anxiety, have increased amid the COVID-19 pandemic (Ahorsu et al., 2020; Courtney et al., 2020; Khoury et al., 2021). Research has shown that isolated learning at home through online teaching during the pandemic has weakened the ability of schools to provide mental health care services to students in a timely manner (Golberstein et al., 2020). In the face of competitive college entrance examinations, high school students are under tremendous academic pressure, and home quarantine and online learning at this stage may have severe psychological effects on students. In addition, teenagers are in transition from being immature to mature persons, and their emotions are direct, solid, and unstable (Casian et al., 2017). Therefore, emotions altered by the pandemic will affect students' studies and may seriously affect their mental health (Yao, 2020). Emotionally unstable people are more likely to experience negative emotions, such as anger, depression, and anxiety (Perkins et al., 2015). In addition, individuals with emotional instability manifest low subjective wellbeing, self-efficacy, and self-evaluation, and they use negative problem-solving methods (Diener et al., 1984; Moeller et al., 2022). In order to stop the spread of the pandemic, people must maintain social distancing. A study found that the Movement Control Order (MCO) due to the COVID-19 pandemic has made the overall index of emotional stability rise from 57 to 95%, indicating that people are emotionally disturbed with COVID-19 pandemic (Mahmud et al., 2020). Due to the effects of COVID-19 lockdown in sub-Saharan Africa (SSA), more than half (52.2%) of the participants had emotional symptoms such as anxiety, worry, depression and anger (Langsi et al., 2021). Margetić et al. (2022) believed that emotional stability was the most significant predictor of mental health. Therefore, it is imperative to study the emotional stability of high school students against this backdrop.

Psychological resilience, which refers to an individual's ability to cope with various challenges and to flexibly overcome external pressures, may develop with personal growth and change (Ai and Hu, 2016). Resilience has become widely studied in psychology as an individual coping resource to resist external pressure (Ke et al., 2022). A study on Arab youth found that the COVID-19 worry negatively predicts psychological resilience and brings them more mental health disorders (Yildirim et al., 2021). Compared with the period before the outbreak of the COVID-19 pandemic, people's psychological resilience has been seriously damaged after the outbreak of the COVID-19 pandemic. However, individuals with strong psychological resilience are better than those with poor psychological resilience in emotional regulation, life satisfaction, subjective wellbeing and overall mental health (Gundogan, 2021; Peker and Cengiz, 2021; Hatun and Kurtça, 2022). It is demonstrated that psychological resilience can mitigate the threat of the pandemic and sustain mental health (Sugawara et al., 2021). Studies have shown that psychological resilience is a significant predictor of positive emotions, with higher psychological resilience levels associated with greater positive emotions (Ong et al., 2010; Yang et al., 2020). Ong et al. (2010) examined the association between psychological resilience and positive emotions and noted that highly resilient individuals show more extraordinarily positive emotions. Moreover, a study using moderated mediation analysis showed that compared with having a higher level of resilience, having a lower level of resilience led to more substantial impacts on emotional disorders (Poole et al., 2017). Work by Zhang et al. (2021a) highlighted that strengthening the emotional regulation of left-behind children promotes psychological resilience. Taken together, these results indicate that individual resilience is positively correlated with emotional stability. Therefore, the first of the three hypotheses that we tested in the present study was that psychological resilience serves as a positive predictor of the emotional stability of high school students.

Mood states, that is, persistent and weak emotional states, may be positive or negative. The theory of the expansion and construction of a positive state of mood holds that positive mood states can expand an individual's actions and thoughts so as to help the individual establish and develop personal resources. In turn, the development of unique resources helps the individual better cope with life challenges (Fredrickson, 2001). The results of a study by Zautra et al. (2005) led to the conclusion that positive and negative moods are mutually independent. Still, when an individual encounters a stressful event, positive moods may greatly reduce negative moods. A positive mood may enhance the resistance to stress and pressure by interrupting the negative mood experience caused by tension or strain to improve an individual's adaptability. Kuang et al. (2019) proposed that a positive emotional contagion increases positive mood states and decreases negative mood states. Positive mood states represent calm and stable emotional states and can facilitate the construction of positive emotions (Xi et al., 2021). Individuals with stable mood states maintain higher psychological resilience and gain more positive emotional experiences. In addition, people with high psychological resilience have the ability to control their emotions well and to maintain a relatively stable state of mind during a stress response. Therefore, the second of the three hypotheses that we tested in this study was that a positive mood state plays a mediating role in the effect of psychological resilience on emotional stability.

Experts believe that the best defense against COVID-19 pandemic is social distancing, although this way can improve physical health, long-term lack of communication, social networking, and sport has a negative impact on mood (Zhang et al., 2021b). The United Kingdom government instigated a societal lockdown in response to the COVID-19 pandemic, Ingram et al. (2020) found that this lockdown brought more negative moods to the British people. Researchers have suggested that negative moods lead to unfavorable performance and that emotional stability attenuates the positive relationship between negative mood and unfavorable performance (Chi et al., 2015). Emotional stability is the best predictor of negative mood (Kardum et al., 2007). Individuals with negative moods have a preference for sad attention, perception, and judgment of emotional information and tend to extract painful information from memories (Niedenthal and Setterlund, 1994). People with negative moods have low (negative) emotional levels when they encounter stressful events, such as pressure and frustration. In addition, they are prone to be affected by extreme emotional reactions, which are difficult to control. People with low psychological resilience tend to have negative mood states when they experience negative life events (Li et al., 2020). A significant negative correlation was found between psychological resilience and negative mood states (Mitchell and Stulhofer, 2021). Therefore, the third of the three hypotheses that we tested in this study was that negative mood state plays a mediating role in the relationship between resilience and emotional stability.

**Figure 1:**
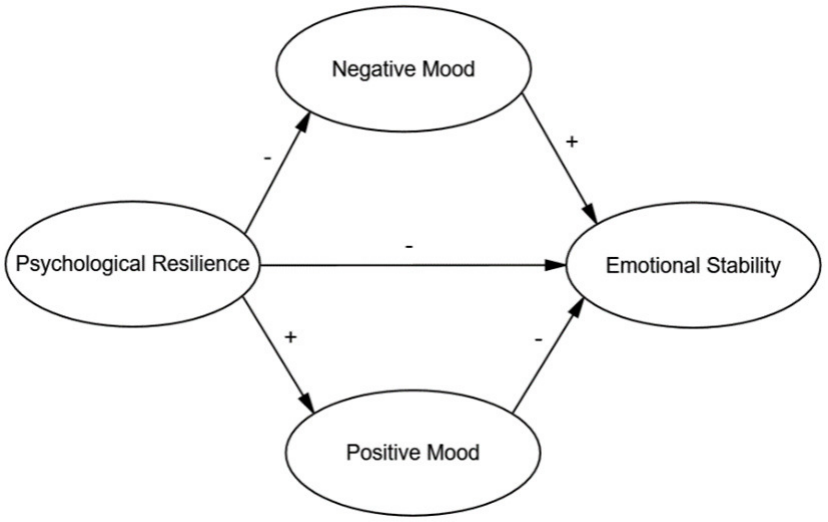
Hypothetical model of the relationship between psychological resilience and emotional stability of high school students. The minus sign between psychological resilience and negative mood indicates that psychological resilience negatively affects the negative mood state; thus higher levels of individual psychological resilience are associated with less negative mood states. The positive sign between psychological resilience and positive mood indicates that psychological resilience positively affects positive mood states; thus, higher levels of individual psychological resilience are associated with more positive emotions. Psychological resilience has a negative impact on emotional stability; thus the higher the level of individual psychological resilience, the more stable the emotions are. Negative mood states positively affect emotional stability; thus the more negative moods an individual has, the more unstable the individual's emotions are. Positive mood negatively affects emotional stability; thus, the more positive moods an individual has, the more stable the individual's emotions will be.

To sum up, there is a close relationship between psychological resilience, mood state and emotional stability. However, the mediating effect of negative mood state and positive mood state on psychological resilience and emotional stability has not been studied by researchers. Moreover, emotional stability is the core standard to measure individual mental health, and the mental health of high school students was seriously affected during the COVID-19 pandemic. Therefore, the study on the emotional stability of high school students is of great help to enrich the theory in the field of psychology. Thus, this study explored the mediating effects of positive and negative mood states on emotional stability by analyzing the predictive impact of psychological resilience on the emotional stability of high school students. Our hypothetical working model describing the internal mechanisms influencing the effects of psychological resilience on emotional stability is shown in Figure 1.

## Materials and methods

### Participants and procedures

A total of 408 students were selected by cluster sampling from the first through third years of a senior high school in Changzhou, Jiangsu, China, From April 11 to April 22, 2020. The study was approved by the Ethics Committee of Medical College of Yangzhou University. With the consent of school leaders and teachers, the face-to-face questionnaires were distributed. After the participants signed the informed consent form, the questionnaire were filled in, and then it was recycled. Questionnaires that did not meet our requirements (such as having incomplete factual data, patterned selection of items, or too many missing items) were excluded, leaving 360 valid questionnaires for our analyses. The effective recovery rate was 88.2%. The mean age of the included students was 16.89 (SD = 0.85) years. The sample comprised 189 boys and 171 girls.

### Measures

#### Psychological resilience scale

We used the psychological resilience scale originally developed by Connor (2003) and modified for Chinese participants by Yu and Zhang (2007). The scale was designed to assess an individual's ability to successfully cope with difficulties or adversity. It included 25 items (e.g., “Under pressure, I can focus and think clearly”) and assessed three factors: tenacity, strength, and optimism. The participants responses were rated on a 5-point Likert scale, ranging from 1 (never) to 5 (all the time), and the total score for each item was calculated. Higher scores indicated higher psychological resilience levels. In this study, the reliability of this scale (Cronbach's α) was 0.941. The results of our confirmatory factor analysis showed that the model was well fit (χ^2^/df = 2.664; comparative fit index [CFI] = 0.92; Tucker-Lewis index [TLI] = 0.91; incremental fit index [IFI] = 0.92; and root mean square error of approximation [RMSEA] = 0.068).

#### Mood state scale

Mood state was measured using the Profile of Mood State scale developed by Zhu (1995). The scale consisted of five negative dimensions (nervousness, anger, fatigue, depression, and panic) and two positive dimensions (energy and self-esteem). Responses were rated on a five-point Likert scale, ranging from 0 to 4. In this study, the reliability of this scale (Cronbach's α) was 0.937. The results of our confirmatory factor analysis showed that the model was well fit (χ^2^/df = 2.535; CFI = 0.91; TLI = 0.90; IFI = 0.91; and RMSEA = 0.065).

#### Emotional stability scale

We used the Eysenck Personality Questionnaire Short Scale in Chinese to assess emotional stability (Qian et al., 2000). The scale had 12 items (e.g., “Do you consider yourself a timid and insecure person?”). Individuals who responded “yes” received one point, and those responding “no” received zero points. Thus, it is important to note that higher scores indicated lower emotional stability, whereas lower scores indicated greater emotional stability. In this study, the reliability of this scale (Cronbach's α) was 0.864. The results of our confirmatory factor analysis showed that the model was well fit (χ^2^/df = 2.533; CFI = 0.93; TLI = 0.92; IFI = 0.93; and RMSEA = 0.065).

### Statistical analysis

The analyses of the relationships were conducted in two steps. First, correlations between psychological resilience, mood state, and emotional stability were calculated in SPSS 23.0. Then, with maximum likelihood estimations, structural equation modeling was used to determine the degree to which mood states mediated the relationship between psychological resilience and emotional stability. This analysis was conducted with SPSS 23.0 AMOS 23.0 software. Model fit was accessed using the comparative fit index [CFI], Tucker-Lewis index [TLI], incremental fit index [IFI], root mean square error of approximation [RMSEA], and CMIN/DF [χ^2^/df]. Modle fit is suggested to be acceptable when CFI ≥ 0.90, TLI ≥ 0.90, IFI ≥ 0.90, RMESA ≤ 0.08, and 1 < χ^2^/df < 3 (Little and Card, 2013).

#### Common method variance

Before analyzing the data, we performed a test for common method bias. The results of our Harman single-factor analysis showed that 11 factors with characteristic roots >1 were extracted from the unrotated exploratory factor analysis, and the maximum factor variance explanation rate was 30.38%, which was lower than the critical standard of 40%. Therefore, there was no common methodological bias in this study. Pearson correlation analysis was then used to assess the correlation between variables in the formal analysis. The latent variable structural equation model was then used to assess the hypothetical mediation effect model.

## Results

### Descriptive statistics and correlation analysis

Means, standard deviations, and correlations among the studied variables are reported in Table 1. Negative mood was negatively correlated with psychological resilience (*r* = −0.57) but was positively correlated with emotional stability (*r* = 0.53). In contrast, positive mood was positively correlated with psychological resilience (*r* = 0.23) but was negatively correlated with emotional stability (*r* = −0.31). There was also a significant negative correlation between psychological resilience and emotional stability (−0.46).

**Table 1:** Descriptive statistics and correlation analysis of negative mood, positive mood, psychological resilience, and emotional stability (*N* = 360).

**Variable**	**Mean ±SD**	**1**	**2**	**3**	**4**
1. Negative mood	24.34 ± 17.81	1			
2. Positive mood	18.35 ± 8.57	−0.079	1		
3. Psychological resilience	71.01 ± 16.00	−0.571**	0.228**	1	
4. Emotional stability	50.05 ± 9.59	0.533**	−0.310**	−0.457**	1

^**^p < 0.01.

### Mediating effect analysis

The model included four latent variables, psychological resilience, negative mood state, positive mood state, and emotional stability, and was well fit (χ^2^/df = 2.209; CFI = 0.95; TLI = 0.94; IFI = 0.95; RMSEA = 0.058). Path analysis of the model (Table 2) indicated that psychological resilience negatively predicted negative mood states (β = −0.6, *p* < 0.001) but positively predicted positive mood states (β = 0.24, *p* < 0.001). Negative mood state positively predicted emotional stability (β = 0.45, *p* < 0.001). Positive mood state negatively predicted emotional stability (β = −0.28, *p* < 0.001). Psychological resilience also negatively predicted emotional stability (β = −0.17, *p* = 0.008). These results indicated that the predictive effect of psychological resilience on the emotional stability of high school students was mediated in part by negative and positive mood states.

**Table 2:** Path analysis.

	**SC**	**SE**	**CR**	***p*-value**
Psychological resilience → Negative mood	−0.600	0.094	−11.738	***
Psychological resilience → Positive mood	0.244	0.095	4.614	***
Negative mood → Emotional stability	0.449	0.005	6.507	***
Positive mood → Emotional stability	−0.275	0.004	−4.98	***
Psychological resilience → Emotional stability	−0.166	0.009	−2.636	0.008

SC, standardization coefficient; SE, standard error; and CR, critical ratio.

^***^p < 0.001.

**Figure 2:**
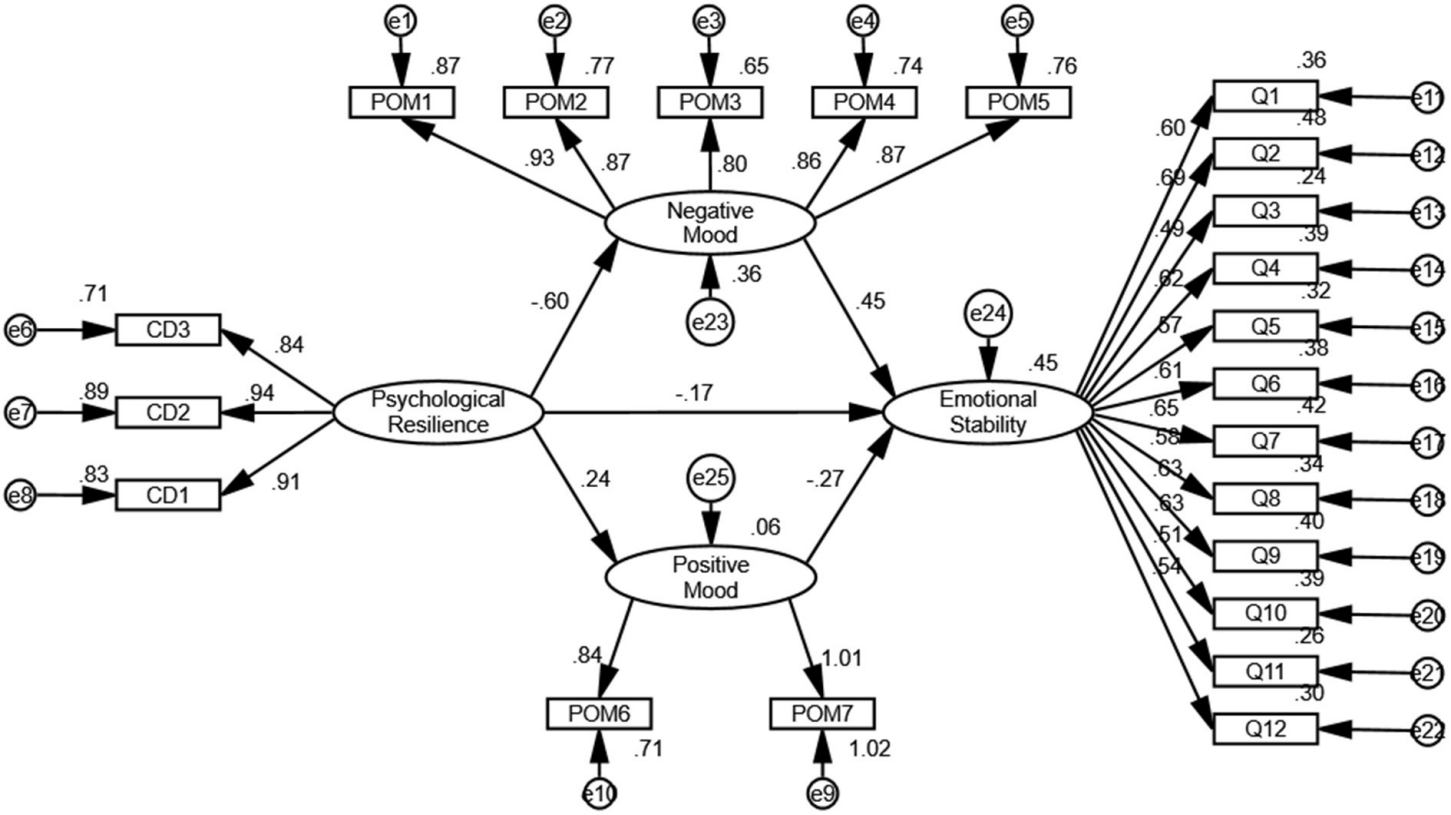
Indirect prediction of mood state on emotional stability of high school students and parallel mediation model examination of negative mood state and positive mood state. Values next to arrows indicate standardized path coefficients. POM1–7 represents nervousness, anger, fatigue, depression, panic, energy and self-esteem; CD1–3, tenacity, strength and optimism; e1–25, residuals; Q1–12, questions 1 through 12 on the scale.

We used the bootstrap method to assess the significance of the bias-corrected mediation effect (Figure 2; Table 3). Because the 95% confidence intervals of the estimated indirect effects did not contain zero, the parallel mediating effects of negative and positive mood states on the relationship between psychological resilience and emotional stability were deemed statistically significant. The indirect effects of negative mood state accounted for 53.6% of the total effect, and the indirect effects of positive mood state accounted for 13.3% of the total effect. Thus, 53.6% of the predicted effect of mood state on the emotional stability of senior high school students was mediated by negative mood states, and 13.3% by positive mood states.

**Table 3:** Mediating effects of positive and negative mood states on the relationship between psychological resilience and emotional stability.

				**Bias-corrected 95% CI**	
**Path**	**Effect value**	**Effect size**	**SE**	**Lower**	**Upper**
Psychological resilience → Negative mood → Emotional stability	−0.269	53.6%	0.044	−0.364	−0.188
Psychological resilience → Positive mood → Emotional stability	−0.067	13.3%	0.021	−0.114	−0.032
Psychological resilience → Emotional stability	−0.166	33.1%	0.074	−0.309	−0.019
Total effect	−0.502	100%	0.049	−0.591	−0.399

CI, confidence interval; SE, standard error.

## Discussion

The results of this study further our understanding of the mediating role of mood states on the relationship between psychological resilience and emotional stability during the COVID-19 pandemic. We found that psychological resilience was negatively correlated with emotional stability and that positive and negative mood states mediated this relationship. The ratio of the negative mood path to the total effect size was much larger than that of ratio of the positive mood path to the total effect size.

This study was conducted to assess psychological resilience and emotional stability among high school students as they experienced home quarantine and returned to school during the COVID-19 pandemic. Our results were consistent with previous studies (Ong et al., 2010). By comparing the psychological resilience norm of people in China, we found that the average psychological resilience score of high school students after home quarantine (71.01; SD, 16.0) was higher than the national norm (65.4; SD, 13.9) (Yu and Zhang, 2007), suggesting that the psychological resilience of the high school students participating in this study was at a good level. A higher level of psychological resilience suggests a higher level of internal homeostasis. When individuals with a high level of internal homeostasis face stress or stressful events, they may have a strong ability to adapt and maintain their emotions in a controllable range, avoiding extreme emotional reactions. However, if this process fails, individuals may passively adapt to the challenge, leading to lower level and even problematic adaptation. This explains why negative mood states accounted for a much larger proportion of the mediating effect than positive mood states on the relationship between resilience and emotional stability in the present study. Our results indicated that psychological resilience not only *directly* affected the emotional stability of high school students, but also *indirectly* affected their emotional stability, which may inform educators seeking to help students with emotional stability.

The present study found that negative and positive mood states played a partial mediating role in predicting the effect of resilience on emotional stability. Compared with the mean score of the revised Chinese scale established by Zhu (1995) for the national norm (102.6; SD, 17.43), the score for the mood state of high school students who returned to school after quarantine was higher (108.7; SD, 21.35), reflecting lower mood states. This finding may be because the high school students in the present study were facing a major turning point in their life—the college entrance examination—while they were also under great psychological pressure due to the COVID-19 pandemic. Thus, students were learning online rather than experiencing their familiar campus life, which may have contributed to an inability to adapt well to online learning. These results indicate that it is particularly important to pay attention to changes in mood states of senior high school students during and after home quarantine.

With the continual changes in society due to the ongoing pandemic, senior high school students are faced with more challenges and are at greater risk of various psychological problems (Lee et al., 2021; Ro et al., 2021; Jardon and Choi, 2022). Given this context, it is particularly important to cultivate the emotional stability of these students. In the present study, we found that psychological resilience may play an significant role in predicting emotional stability among senior high school students, which can be realized through the mediating effect of mood states. Adolescence is a key period for cultivating personality and self-concept, and the cultivation of emotional stability is of great importance for self-improvement. Thus, educators should pay attention to the emotional stability of senior high school students and provide psychological intervention when necessary. In doing so, they may assist students in the development of greater emotional stability, promoting emotional stability in the face of setbacks, increased pressure, stress, and other events.

### Limitations and future research directions

This study had some limitations. First, this study used a cross-sectional design to explore the relationships between various variables. Future longitudinal studies should be used to further analyze the relationships among psychological resilience, mood state, and emotional stability, and explore whether it can promote the growth of these three indicators of high school students through sports intervention in the future. Second, whether mood state indeed plays a moderating role between psychological resilience and emotional stability will require further study. Third, the specific processes underlying the influence of resilience on mood state and emotional stability should be further explored, such as the influences of tension, fatigue, depression, strength, optimism, and tenacity. The combination of the present experimental method using questionnaire survey data analyses will be useful in future research to further explore the emotional stability of high school students. Fourth, due to the impact of COVID-19 pandemic, the sample size of this study is relatively small, so we should expand the sample size to supplement and explain the results of this study. Fifth, the quantitative information of high school students exposed to the COVID-19 pandemic has not been collected. In the future, more accurate results can be obtained by comparing with high school students unaffected by the COVID-19 pandemic.

## Conclusion

The present study found that psychological resilience was a positive predictor of emotional stability in high school students through both direct and indirect effects. The effects of psychological resilience on the emotional stability of senior high school students were realized through two mediating pathways: negative mood states and positive mood states, and the mediating effect of negative mood states was greater than that of positive mood states.

## Data availability statement

The original contributions presented in the study are included in the article/Supplementary material, further inquiries can be directed to the corresponding author.

## Ethics statement

The studies involving human participants were reviewed and approved by Ethics Committee of Medical College of Yangzhou University. Written informed consent to participate in this study was provided by the participants' legal guardian/next of kin.

## Author contributions

QW contributed to conception and design of the study, polished, and modified the manuscript. FH organized the database, performed the statistical analysis, and wrote the first draft of the manuscript. All authors contributed to the article and approved the submitted version.

## Conflict of interest

The authors declare that the research was conducted in the absence of any commercial or financial relationships that could be construed as a potential conflict of interest.

## Publisher's note

All claims expressed in this article are solely those of the authors and do not necessarily represent those of their affiliated organizations, or those of the publisher, the editors and the reviewers. Any product that may be evaluated in this article, or claim that may be made by its manufacturer, is not guaranteed or endorsed by the publisher.
